# Principles for data analysis workflows

**DOI:** 10.1371/journal.pcbi.1008770

**Published:** 2021-03-18

**Authors:** Sara Stoudt, Váleri N. Vásquez, Ciera C. Martinez

**Affiliations:** 1 Berkeley Institute for Data Science, University of California Berkeley, Berkeley, California, United States of America; 2 Statistical & Data Sciences Program, Smith College, Northampton, Massachusetts, United States of America; 3 Energy and Resources Group, University of California Berkeley, Berkeley, California, United States of America; 4 Department of Molecular and Cellular Biology, University of California Berkeley, Berkeley, California, United States of America; SIB Swiss Institute of Bioinformatics, SWITZERLAND

## Abstract

A systematic and reproducible “workflow”—the process that moves a scientific investigation from raw data to coherent research question to insightful contribution—should be a fundamental part of academic data-intensive research practice. In this paper, we elaborate basic principles of a reproducible data analysis workflow by defining 3 phases: the Explore, Refine, and Produce Phases. Each phase is roughly centered around the audience to whom research decisions, methodologies, and results are being immediately communicated. Importantly, each phase can also give rise to a number of research products beyond traditional academic publications. Where relevant, we draw analogies between design principles and established practice in software development. The guidance provided here is not intended to be a strict rulebook; rather, the suggestions for practices and tools to advance reproducible, sound data-intensive analysis may furnish support for both students new to research and current researchers who are new to data-intensive work.

## Introduction

Both traditional science fields and the humanities are becoming increasingly data driven and computational. Researchers who may not identify as data scientists are working with large and complex data on a regular basis. A systematic and reproducible **research workflow**—the process that moves a scientific investigation from raw data to coherent research question to insightful contribution—should be a fundamental part of data-intensive research practice in any academic discipline. The importance and effective development of a workflow should, in turn, be a cornerstone of the data science education designed to prepare researchers across disciplinary specializations.

Data science education tends to review foundational statistical analysis methods [1] and furnish training in **computational tools**, software, and programming languages. In scientific fields, education and training includes a review of domain-specific methods and tools, but generally omits guidance on the coding practices relevant to developing new analysis software—a skill of growing relevance in data-intensive scientific fields [2]. Meanwhile, the holistic discussion of how to develop and pursue a research workflow is often left out of introductions to both data science and disciplinary science. Too frequently, students and academic practitioners of data-intensive research are left to learn these essential skills on their own and on the job. Guidance on the breadth of potential products that can emerge from research is also lacking. In the interest of both **reproducible** science (providing the necessary data and code to recreate the results) and effective career building, researchers should be primed to regularly generate outputs over the course of their workflow.

The goal of this paper is to deconstruct an academic **data-intensive research** project, demonstrating how both design principles and software development methods can motivate the creation and standardization of practices for reproducible data and code. The implementation of such practices generates research products that can be effectively communicated, in addition to constituting a scientific contribution. Here, “data-intensive” research is used interchangeably with “data science” in a recognition of the breadth of domain applications that draw upon computational analysis methods and workflows. (We define other terms we’ve bolded throughout this paper in Box 1). To be useful, let alone high impact, research analyses should be contextualized in the data processing decisions that led to their creation and accompanied by a narrative that explains why the rest of the world should be interested. One way of thinking about this is that the scientific method should be tangibly reflected, and feasibly reproducible, in any data-intensive research project.

**Box 1. Terminology**

This box provides definitions for terms in **bold** throughout the text. Terms are sorted alphabetically and cross referenced where applicable.

**Agile:** An iterative software development framework which adheres to the principles described in the Manifesto for Agile software development [35] (e.g., breaks up work into small increments).

**Accessor function:** A function that returns the value of a variable (synonymous term: getter function).

**Assertion:** An expression that is expected to be true at a particular point in the code.

**Computational tool:** May include libraries, packages, collections of functions, and/or data structures that have been consciously designed to facilitate the development and pursuit of data-intensive questions (synonymous term: software tool).

**Continuous integration:** Automatic tests that updated code.

**Gut check:** Also “data gut check.” Quick, broad, and shallow testing [48] before and during data analysis. Although this is usually described in the context of software development, the concept of a data-specific gut check can include checking the dimensions of data structures after merging or assessing null values/missing values, zero values, negative values, and ranges of values to see if they make sense (synonymous words: smoke test, sanity check [49], consistency check, sniff test, soundness check).

**Data-intensive research**: Research that is centrally based on the analysis of data and its structural or statistical properties. May include but is not limited to research that hinges on large volumes of data or a wide variety of data types requiring computational skills to approach such research (synonymous term: data science research). “Data science” as a stand-alone term may also refer more broadly to the use of computational tools and statistical methods to gain insights from digitized information.

**Data structure:** A format for storing data values and definition of operations that can be applied to data of a particular type.

**Defensive programming**: Strategies to guard against failures or bugs in code; this includes the use of tests and assertions.

**Design thinking:** The iterative process of defining a problem then identifying and prototyping potential solutions to that problem, with an emphasis on solutions that are empathetic to the particular needs of the target user.

**Docstring:** A code comment for a particular line of code that describes what a function does, as opposed to how the function performs that operation.

**DOI:** A digital object identifier or DOI is a unique handle, standardized by the International Organization for Standardization (ISO), that can be assigned to different types of information objects.

**Extensibility:** The flexibility to be extended or repurposed in a new scenario.

**Function:** A piece of more abstracted code that can be reused to perform the same operation on different inputs of the same type and has a standardized output [50–52].

**Getter function:** Another term for an accessor function.

**Integrated Development Environment (IDE):** A software application that facilitates software development and minimally consists of a source code editor, build automation tools, and a debugger.

**Modularity:** An ability to separate different functionality into stand-alone pieces.

**Mutator method:** A function used to control changes to variables. See “setter function” and “accessor function.”

**Notebook:** A computational or physical place to store details of a research process including decisions made.

**Mechanistic code**: Code used to perform a task as opposed to conduct an analysis. Examples include processing functions and plotting functions.

**Overwrite:** The process, intentional or accidental, of assigning new values to existing variables.

**Package manager:** A system used to automate the installation and configuration of software.

**Pipeline**: A series of programmatic processes during data analysis and data cleaning, usually linear in nature, that can be automated and usually be described in the context of inputs and outputs.

**Premature optimization**: Focusing on details before the general scheme is decided upon.

**Refactoring:** A change in code, such as file renaming, to make it more organized without changing the overall output or behavior.

**Replicable:** A new study arrives at the same scientific findings as a previous study, collecting new data (with the same or different methods) and completes new analyses [53–55].

**Reproducible:** Authors provide all the necessary data, and the computer codes to run the analysis again, recreating the results [53–55].

**Script**: A collection of code, ideally related to one particular step in the data analysis.

**Setter function:** A type of function that controls changes to variables. It is used to directly access and alter specific values (synonymous term: mutator method).

**Serialization:** The process of saving data structures, inputs and outputs, and experimental setups generally in a storable, shareable format. Serialized information can be reconstructed in different computer environments for the purpose of replicating or reproducing experiments.

**Software development:** A process of writing and documenting code in pursuit of an end goal, typically focused on process over analysis.

**Source code editor:** A program that facilitates changes to code by an author.

**Technical debt:** The extra work you defer by pursuing an easier, yet not ideal solution, early on in the coding process.

**Test-driven development:** Each change in code should be verified against tests to prove its functionality.

**Unit test:** A code test for the smallest chunk of code that is actually testable.

**Version control:** A way of managing changes to code or documentation that maintains a record of changes over time.

**White paper:** An informative, at least semiformal document that explains a particular issue but is not peer reviewed.

**Workflow**: The process that moves a scientific investigation from raw data to coherent research question to insightful contribution. This often involves a complex series of processes and includes a mixture of machine automation and human intervention. It is a nonlinear and iterative exercise.

Discussions of “workflow” in data science can take on many different meanings depending on the context. For example, the term “workflow” often gets conflated with the term “**pipeline**” in the context of software development and engineering. Pipelines are often described as a series of processes that can be programmatically defined and automated and explained in the context of inputs and outputs. However, in this paper, we offer an important distinction between pipelines and workflows: The former refers to what a computer does, for example, when a piece of software automatically runs a series of Bash or R **script**s. For the purpose of this paper, a workflow describes what a researcher does to make advances on scientific questions: developing hypotheses, wrangling data, writing code, and interpreting results.

Data analysis workflows can culminate in a number of outcomes that are not restricted to the traditional products of software engineering (software tools and packages) or academia (research papers). Rather, the workflow that a researcher defines and iterates over the course of a data science project can lead to intellectual contributions as varied as novel data sets, new methodological approaches, or teaching materials in addition to the classical tools, packages, and papers. While the workflow should be designed to serve the researcher and their collaborators, maintaining a structured approach throughout the process will inform results that are **replicable** (see replicable versus reproducible in Box 1) and easily translated into a variety of products that furnish scientific insights for broader consumption.

In the following sections, we explain the basic principles of a constructive and productive data analysis workflow by defining 3 phases: the Explore, Refine, and Produce Phases. Each phase is roughly centered around the audience to whom research decisions, methodologies, and results are being immediately communicated. Where relevant, we draw analogies to the realm of **design thinking** and **software development**. While the 3 phases described here are not intended to be a strict rulebook, we hope that the many references to additional resources—and suggestions for nontraditional research products—provide guidance and support for both students new to research and current researchers who are new to data-intensive work.

## The Explore, Refine, Produce (ERP) workflow for data-intensive research

**Fig 1:**
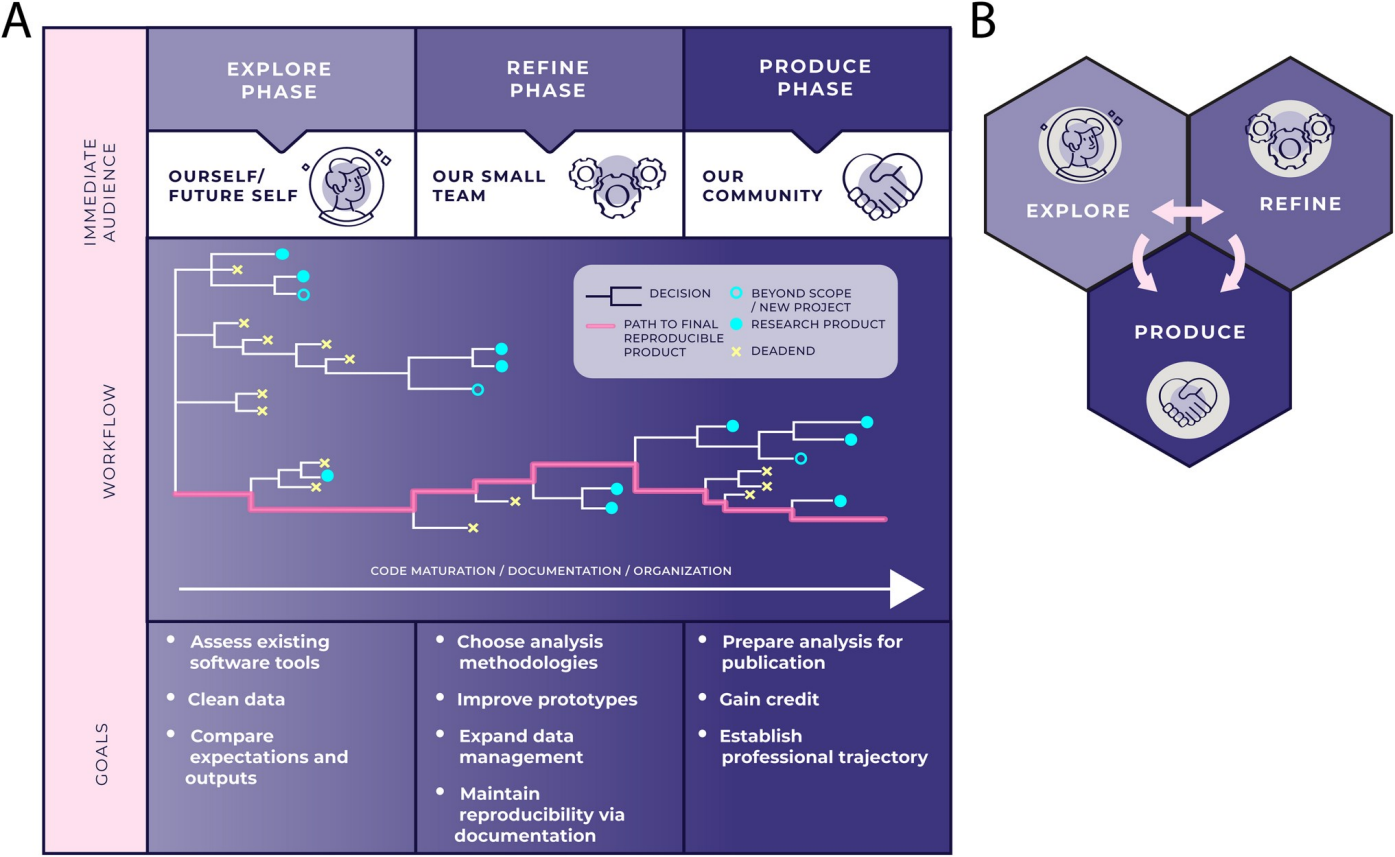
ERP workflow for data-intensive research. (A) We deconstruct a data-intensive research project into 3 phases, visualizing this process as a tree structure. Each branch in the tree represents a decision that needs to be made about the project, such as data cleaning, refining the scope of the research, or using a particular tool or model. Throughout the natural life of a project, there are many dead ends (yellow Xs). These may include choices that do not work, such as experimentation with a tool that is ultimately not compatible with our data. Dead ends can result in informal learning or procedural fine-tuning. Some dead ends that lie beyond the scope of our current project may turn into a new project later on (open turquoise circles). Throughout the Explore and Refine Phases, we are concurrently in the Produce Phase because research products (closed turquoise circles) can arise at any point throughout the workflow. Products, regardless of the phase that generates their content, contribute to scientific understanding and advance the researcher’s career goals. Thus, the data-intensive research portfolio and corresponding academic CV can be grown at any point in the workflow. (B) The ERP workflow as a nonlinear cycle. Although the tree diagram displayed in Fig 1A accurately depicts the many choices and dead ends that a research project contains, it does not as easily reflect the nonlinearity of the process; Fig 1B’s representation aims to fill this gap. We often iterate between the Explore and Refine Phases while concurrently contributing content to the Produce Phase. The time spent in each phase can vary significantly across different types of projects. For example, hypothesis generation in the Explore Phase might be the biggest hurdle in one project, while effectively communicating a result to a broader audience in the Produce Phase might be the most challenging aspect of another project.

We partition the workflow of a data-intensive research process into 3 phases: Explore, Refine, and Produce. These phases, collectively the ERP workflow, are visually described in Fig 1A and 1B. In the Explore Phase, researchers “meet” their data: process it, interrogate it, and sift through potential solutions to a problem of interest. In the Refine Phase, researchers narrow their focus to a particularly promising approach, develop prototypes, and organize their code into a clearer narrative. The Produce Phase happens concurrently with the Explore and Refine Phases. In this phase, researchers prepare their work for broader consumption and critique.

Each phase has an immediate audience—the researcher themselves, their collaborative groups, or the public—that broadens progressively and guides priorities. Each of the 3 phases can benefit from standards that the software development community uses to streamline their code-based pipelines, as well as from principles the design community uses to generate and carry out ideas; many such practices can be adapted to help structure a data-intensive researcher’s workflow. The Explore and Refine Phases provide fodder for the concurrent Produce Phase. We hope that the potential to produce a variety of research products throughout a data-intensive research process, rather than merely at the end of a project, motivates researchers to apply the ERP workflow.

## Phase 1: Explore

Data-intensive research projects typically start with a domain-specific question or a particular data set to explore [3]. There is no fixed, cross-disciplinary rule that defines the point in a workflow by which a hypothesis must be established. This paper adopts an open-minded approach concerning the timing of hypothesis generation [4], assuming that data-intensive research projects can be motivated by either an explicit, preexisting hypothesis or a new data set about which no strong preconceived assumptions or intuitions exist. The often messy Explore Phase is rarely discussed as an explicit step of the methodological process, but it is an essential component of research: It allows us to gain intuition about our data, informing future phases of the workflow. As we explore our data, we refine our research question and work toward the articulation of a well-defined problem. The following section will address how to reap the benefits of data set and problem space exploration and provide pointers on how to impose structure and reproducibility during this inherently creative phase of the research workflow.

### Designing data analysis: Goals and standards of the Explore Phase

Trial and error is the hallmark of the Explore Phase (note the density of “deadends” and decisions made in this phase in Fig 1A). In “Designerly Ways of Knowing” [5], the design process is described as a “co-evolution of solution and problem spaces.” Like designers, data-intensive researchers explore the problem space, learn about the potential structure of the solution space, and iterate between the 2 spaces. Importantly, the difficulties we encounter in this phase help us build empathy for an eventual audience beyond ourselves. It is here that we experience firsthand the challenges of processing our data set, framing domain research questions appropriate to it, and structuring the beginnings of a workflow. Documenting our trial and error helps our own work stay on track in addition to assisting future researchers facing similar challenges.

One end goal of the Explore Phase is to determine whether new questions of interest might be answered by leveraging existing software tools (either off the shelf or with minor adjustments), rather than building new computational capabilities ourselves. For example, during this phase, a common activity includes surveying the software available for our data set or problem space and estimating its utility for the unique demands of our current analysis. Through exploration, we learn about relevant computational and analysis tools while concurrently building an understanding of our data.

A second important goal of the Explore Phase is data cleaning and developing a strategy to analyze our data. This is a dynamic process that often goes hand in hand with improving our understanding of the data. During the Explore Phase, we redesign and reformat data structures, identify important variables, remove redundancies, take note of missing information, and ponder outliers in our data set. Once we have established the software tools—the programming language, data analysis packages, and a handful of the useful **functions** therein—that are best suited to our data and domain area, we also start putting those tools to use [6]. In addition, during the Explore Phase, we perform initial tests, build a simple model, or create some basic visualizations to better grasp the contents of our data set and check for expected outputs. Our research is underway in earnest now, and this effort will help us to identify what questions we might be able to ask of our data.

The Explore Phase is often a solo endeavor; as shown in Fig 1A, our audience is typically our current or future self. This can make navigating the phase difficult, especially for new researchers. It also complicates a third goal of this phase: documentation. In this phase, we ourselves are our only audience, and if we are not conscientious documenters, we can easily end up concluding the phase without the ability to coherently describe our research process up to that point. Record keeping in the Explore Phase is often subject to our individual style of approaching problems. Some styles work in real time, subsetting or reconfiguring data as ideas occur. More methodical styles tend to systematically plan exploratory steps, recording them before taking action. These natural tendencies impact the state of our analysis code, affecting its readability and reproducibility.

However, there are strategies—inspired by analogous software development principles—that can help set us up for success in meeting the standards of reproducibility [7] relevant to a scientifically sound research workflow. These strategies impose a semblance of order on the Explore Phase. To avoid concerns of **premature optimization** [8] while we are iterating during this phase, documentation is the primary goal, rather than fine-tuning the code structure and style. Documentation enables the traceability of a researcher’s workflow, such that all efforts are replicable and final outcomes are reproducible.

### Analogies to software development in the Explore Phase

#### Documentation: Code and process

Software engineers typically value formal documentation that is readable by software users. While the audience for our data analysis code may not be defined as a software user per se, documentation is still vital for workflow development. Documentation for data analysis workflows can come in many forms, including comments describing individual lines of code, README files orienting a reader within a code repository, descriptive commit history logs tracking the progress of code development, **docstrings** detailing function capabilities, and vignettes providing example applications. Documentation provides both a user manual for particular tools within a project (for example, data cleaning functions), and a reference log describing scientific research decisions and their rationale (for example, the reasons behind specific parameter choices).

In the Explore Phase, we may identify with the type of programmer described by Brant and colleagues as “opportunistic” [9]. This type of programmer finds it challenging to prioritize documenting and organizing code that they see as impermanent or a work in progress. “Opportunistic” programmers tend to build code using others’ tools, focusing on writing “glue” code that links preexisting components and iterate quickly. Hartmann and colleagues also describe this mash-up approach [10]. Rather than “opportunistic programmers,” their study focuses on “opportunistic designers.” This style of design “search[es] for bridges,” finding connections between what first appears to be different fields. Data-intensive researchers often use existing tools to answer questions of interest; we tend to build our own only when needed.

Even if the code that is used for data exploration is not developed into a software-based final research product, the exploratory process as a whole should exist as a permanent record: Future scientists should be able to rerun our analysis and work from where we left off, beginning from raw, unprocessed data. Therefore, documenting choices and decisions we make along the way is crucial to making sure we do not forget any aspect of the analysis workflow, because each choice may ultimately impact the final results. For example, if we remove some data points from our analyses, we should know which data points we removed—and our reason for removing them—and be able to communicate those choices when we start sharing our work with others. This is an important argument against ephemerally conducting our data analysis work via the command line.

Instead of the command line, tools like a computational **notebook** [11] can help capture a researcher’s decision-making process in real time [12]. A computational notebook where we never delete code, and—to avoid overwriting named variables—only move forward in our document, could act as “version control designed for a 10-minute scale” that Brant and colleagues found might help the “opportunistic” programmer. More recent advances in this area include the reactive notebook [13–14]. Such tools assist documentation while potentially enhancing our creativity during the Explore Phase. The bare minimum documentation of our Explore Phase might therefore include such a notebook or an annotated script [15] to record all analyses that we perform and code that we write.

To go a step beyond annotated scripts or notebooks, researchers might employ a **version control** system such as Git. With its issues, branches, and informative commit messages, Git is another useful way to maintain a record of our trial-and-error process and track which files are progressing toward which goals of the overall project. Using Git together with a public online hosting service such as GitHub allows us to share our work with collaborators and the public in real time, if we so choose.

A researcher dedicated to conducting an even more thoroughly documented Explore Phase may take Ford’s advice and include notes that explicitly document our stream of consciousness [16]. Our notes should be able to efficiently convey what failed, what worked but was uninteresting or beyond scope of the project, and what paths of inquiry we will continue forward with in more depth (Fig 1A). In this way, as we transition from the Explore Phase to the Refine Phase, we will have some signposts to guide our way.

#### Testing: Comparing expectations to output

As Ford [16] explains, we face competing goals in the Explore Phase: We want to get results quickly, but we also want to be confident in our answers. Her strategy is to focus on documentation over tests for one-off analyses that will not form part of a larger research project. However, the complete absence of formal tests may raise a red flag for some data scientists used to the concept of **test-driven development**. This is a tension between the code-based work conducted in scientific research versus software development: Tests help build confidence in analysis code and convince users that it is reliable or accurate, but tests also imply finality and take time to write that we may not be willing to allocate in the experimental Explore Phase. However, software development style tests do have useful analogs in data analysis efforts: We can think of tests, in the data analysis sense, as a way of checking whether our expectations match the reality of a piece of code’s output.

Imagine we are looking at a data set for the first time. What weird things can happen? The type of variable might not be what we expect (for example, the integer 4 instead of the float 4.0). The data set could also include unexpected aspects (for example, dates formatted as strings instead of numbers). The amount of missing data may be larger than we thought, and this missingness could be coded in a variety of ways (for example, as a NaN, NULL, or −999). Finally, the dimensions of a data frame after merging or subsetting it for data cleaning may not match our expectations. Such gaps in expectation versus reality are “silent faults” [17]. Without checking for them explicitly, we might proceed with our analysis unaware that anything is amiss and encode that error in our results.

For these reasons, every data exploration should include quantitative and qualitative “gut checks” [18] that can help us diagnose an expectation mismatch as we go about examining and manipulating our data. We may check assumptions about data quality such as the proportion of missing values, verify that a joined data set has the expected dimensions, or ascertain the statistical distributions of well-known data categories. In this latter case, having domain knowledge can help us understand what to expect. We may want to compare 2 data sets (for example, pre- and post-processed versions) to ensure they are the same [19]; we may also evaluate diagnostic plots to assess a model’s goodness of fit. Each of the elements that gut checks help us monitor will impact the accuracy and direction of our future analyses.

We perform these manual checks to reassure ourselves that our actions at each step of data cleaning, processing, or preliminary analysis worked as expected. However, these types of checks often rely on us as researchers visually assessing output and deciding if we agree with it. As we transition to needing to convince users beyond ourselves of the correctness of our work, we may consider employing **defensive programming** techniques that help guard against specific mistakes. An example of defensive programming in the Julia language is the use of assertions, such as the @assert macro to validate values or function outputs. Another option includes writing “chatty functions” [20] that signal a user to pause, examine the output, and decide if they agree with it.

### When to transition from the Explore Phase: Balancing breadth and depth

A researcher in the Explore Phase experiments with a variety of potential data configurations, analysis tools, and research directions. Not all of these may bear fruit in the form of novel questions or promising preliminary findings. Learning how to find a balance between the breadth and depth of data exploration helps us understand when to transition to the Refine Phase of data-intensive research. Specific questions to ask ourselves as we prepare to transition between the Explore Phase and the Refine Phase can be found in Box 2.

**Box 2. Questions**

This box provides guiding questions to assist readers in navigating through each workflow phase. Questions pertain to planning, organization, and accountability over the course of workflow iteration.

Questions to ask in the Explore Phase

- Who can read through our materials and understand our workflow?
  - Good: Ourselves (e.g., Code includes signposts refreshing our memory of what is happening where.)
  - Better: Our small team who has specialized knowledge about the context of the problem.
  - Best: Anyone with experience using similar tools to us.
- What material do we think is worth continuing into the next phase?
  - Good: Dead ends marked differently than relevant and working code.
  - Better: Material connected to a handful of promising leads.
  - Best: Material connected to a clearly defined scope.
- Where does the code live?
  - Good: Backed up in a second location in addition to our computer.
  - Better: Within a shared space among our team (e.g., Google Drive, Box, etc.).
  - Best: Within a version control system (e.g., GitHub) that furnishes a complete timeline of actions taken.
- Why did we make particular data cleaning and analysis decisions?
  - Good: Noted in a separate place from our code (e.g., a physical notebook).
  - Better: Noted in comments throughout the code itself, with expectations informally checked.
  - Best: Noted systematically throughout code as part of a narrative, with expectations formally checked.

Questions to ask in the Refine Phase

- Who is in our team?
- What are our teammates’ skill levels?
  - Consider career level, computational experience, and domain-specific experience.
- How do we communicate methodology with our teammates’ skills in mind?
- Are there established standards within the team that need to be adopted or conflict with our Explore Phase workflow?
  - What reproducibility tools can be agreed upon?
- What is the main takeaway of our findings?
  - How can our work be packaged into impactful research products?
  - Can we explain the same important results across different platforms (e.g., blog post in addition to white paper)?
- Who will be affected by the outcomes of our work?
  - How can we alert these people and make our work accessible?
- Why is our work important for our domain-specific field? For broader society?
  - How can we use narrative to make this clear?

Questions to ask in the Produce Phase

- Who is the intended audience for our research product(s)?
  - Do we have more than 1 audience?
- What is the next step in our research?
- Where do we plan to publish?
  - Can we turn our work into more than 1 publishable product?
- When do we expect a research product to be ready for a broader audience?
  - Consider products throughout the entire workflow.
- Why should we decide to build a software tool based on our work?
  - See suggestions in the Tool development guide (Box 4).

Imposing structure at certain points throughout the Explore Phase can help to balance our wide search for solutions with our deep dives into particular options. In an analogy to the software development world, we can treat our exploratory code as a code release—the marker of a stable version of a piece of software. For example, we can take stock of the code we have written at set intervals, decide what aspects of the analysis conducted using it seem most promising, and focus our attention on more formally tuning those parts of the code. At this point, we can also note the presence of research “dead ends” and perhaps record where they fit into our thought process. Some trains of thought may not continue into the next phase or become a formal research product, but they can still contribute to our understanding of the problem or eliminate a potential solution from consideration. As the project matures, computational pipelines are established. These inform project workflow, and tools, such as Snakemake and Nextflow, can begin to be used to improve the flexibility and reproducibility of the project [21–23]. As we make decisions about which research direction we are going to pursue, we can also adjust our file structure and organize files into directories with more informative names.

Just as Cross [5] finds that a “reasonably-structured process” leads to design success where “rigid, over-structured approaches” find less success, a balance between the formality of documentation and testing and the informality of creative discovery is key to the Explore Phase of data-intensive research. By taking inspiration from software development and adapting the principles of that arena to fit our data analysis work, we add enough structure to this phase to ease transition into the next phase of the research workflow.

## Phase 2: Refine

Inevitably, we reach a point in the Explore Phase when we have acquainted ourselves with our data set, processed and cleaned it, identified interesting research questions that might be asked using it, and found the analysis tools that we prefer to apply. Having reached this important juncture, we may also wish to expand our audience from ourselves to a team of research collaborators. It is at this point that we are ready to transition to the Refine Phase. However, we should keep in mind that new insights may bring us back to the Explore Phase: Over the lifetime of a given research project, we are likely to cycle through each workflow phase multiple times.

In the Refine Phase, the extension of our target audience demands a higher standard for communicating our research decisions as well as a more formal approach to organizing our workflow and documenting and testing our code. In this section, we will discuss principles for structuring our data analysis in the Refine Phase. This phase will ultimately prepare our work for polishing into more traditional research products, including peer-reviewed academic papers.

### Designing data analysis: Goals and standards of the Refine Phase

The Refine Phase encompasses many critical aspects of a data-intensive research project. Additional data cleaning may be conducted, analysis methodologies are chosen, and the final experimental design is decided upon. Experimental design may include identifying case studies for variables of interest within our data. If applicable, it is during this phase that we determine the details of simulations. Preliminary results from the Explore Phase inform how we might improve upon or scale up prototypes in the Refine Phase. Data management is essential during this phase and can be expanded to include the **serialization** of experimental setups. Finally, standards of reproducibility should be maintained throughout. Each of these aspects constitutes an important goal of the Refine Phase as we determine the most promising avenues for focusing our research workflow en route to the polished research products that will emerge from this phase and demand even higher reproducibility standards.

All of these goals are developed in conjunction with our research team. Therefore, decisions should be documented and communicated in a way that is reproducible and constructive within that group. Just as the solitary nature of the Explore Phase can be daunting, the collaboration that may happen in the Refine Phase brings its own set of challenges as we figure out how to best work together. Our team can be defined as the people who participate in developing the research question, preparing the data set it is applied to, coding the analysis, or interpreting the results. It might also include individuals who offer feedback about the progress of our work. In the context of academia, our team usually includes our laboratory or research group. Like most other aspects of data-intensive research, our team may evolve as the project evolves. But however we define our team, its members inform how our efforts proceed during the Refine Phase: Thus, another primary goal of the Refine Phase is establishing group-based standards for the research workflow. Specific questions to ask ourselves during this phase can be found in Box 2.

In recent years, the conversation on standards within academic data science and scientific computing has shifted from “best” practices [24] to “good enough” practices [25]. This is an important distinction when establishing team standards during the Refine Phase: Reproducibility is a spectrum [26], and collaborative work in data-intensive research carries unique demands on researchers as scholars and coworkers [27]. At this point in the research workflow, standards should be adopted according to their appropriateness for our team. This means talking among ourselves not only about scientific results, but also about the computational experimental design that led to those results and the role that each team member plays in the research workflow. Establishing methods for effective communication is therefore another important goal in the Refine Phase, as we cannot develop group-based standards for the research workflow without it.

### Analogies to software development in the Refine Phase

#### Documentation as a driver of reproducibility

The concept of literate programming [8] is at the core of an effective Refine Phase. This philosophy brings together code with human-readable explanations, allowing scientists to demonstrate the functionality of their code in the context of words and visualizations that describe the rationale for and results of their analysis. The computational notebooks that were useful in the Explore Phase are also applicable here, where they can assist with team-wide discussions, research development, prototyping, and idea sharing. Jupyter Notebooks [28] are agnostic to choice of programming language and so provide a good option for research teams that may be working with a diverse code base or different levels of comfort with a particular programming language. Language-specific interfaces such as R’s RMarkdown functionality [29] and Literate.jl or the reactive notebook put forward by Pluto.jl in the Julia programming language furnish additional options for literate programming.

The same strategies that promote scientific reproducibility for traditional laboratory notebooks can be applied to the computational notebook [30]. After all, our data-intensive research workflow can be considered a sort of scientific experiment—we develop a hypothesis, query our data, support or reject our hypothesis, and state our insights. A central tenet of scientific reproducibility is recording inputs relevant to a given analysis, such as parameter choices, and explaining any calculation used to obtain them so that our outputs can later be verifiably replicated. Methodological details—for example, the decision to develop a dynamic model in continuous time versus discrete time or the choice of a specific statistical analysis over alternative options—should also be fully explained in computational notebooks developed during the Refine Phase. Domain knowledge may inform such decisions, making this an important part of proper notebook documentation; such details should also be elaborated in the final research product. Computational research descriptions in academic journals generally include a narrative relevant to their final results, but these descriptions often do not include enough methodological detail to enable replicability, much less reproducibility. However, this is changing with time [31,32].

As scientists, we should keep a record of the tools we use to obtain our results in addition to our methodological process. In a data-intensive research workflow, this includes documenting the specific version of any software that we used, as well as its relevant dependencies and compatibility constraints. Recording this information at the top of the computational notebook that details our data science experiment allows future researchers—including ourselves and our teams—to establish the precise computational environment that was used to run the original research analysis. Our chosen programming language may supply automated approaches for doing this, such as a **package manager**, simplifying matters and painlessly raising the standards of reproducibility in a research team. The unprecedented levels of reproducibility possible in modern computational environments have produced some variance in the expectations of different research communities; it behooves the research team to investigate the community-level standards applicable to our specific domain science and chosen programming language.

A notebook can include more than a deep dive into a full-fledged data science experiment. It can also involve exploring and communicating basic properties of the data, whether for purposes of training team members new to the project or for brainstorming alternative possible approaches to a piece of research. In the Exploration Phase, we have discovered characteristics of our data that we want our research team to know about, for example, outliers or unexpected distributions, and created preliminary visualizations to better understand their presence. In the Refine Phase, we may choose to improve these initial plots and reprise our data processing decisions with team members to ensure that the logic we applied still holds.

Computational notebooks can live in private or public repositories to ensure accessibility and transparency among team members. A version control system such as Git continues to be broadly useful for documentation purposes in the Refine Phase, beyond acting as a storage site for computational notebooks. Especially as our team and code base grows larger, a history of commits and pull requests helps keep track of responsibilities, coding or data issues, and general workflow.

Importantly however, all tools have their appropriate use cases. Researchers should not develop an overt reliance on any one tool and should learn to recognize when different tools are required. For example, computational notebooks may quickly become unwieldy for certain projects and large teams, incurring **technical debt** in the form of duplications or overwritten variables. As our research project grows in complexity and size, or gains team members, we may want to transition to an **Integrated Development Environment** (IDE) or a **source code editor**—which interact easily with **container** environments like Docker and version control systems such as GitHub—to help scale our data analysis, while retaining important properties like reproducibility.

#### Testing and establishing code modularity

Code in data-intensive research is generally written as a means to an end, the end being a scientific result from which researchers can draw conclusions. This stands in stark contrast to the purpose of code developed by data engineers or computer scientists, which is generally written to optimize a mechanistic function for maximum efficiency. During the Refine Phase, we may find ourselves with both analysis-relevant and **mechanistic code**, especially in “big data” statistical analyses or complex dynamic simulations where optimized computation becomes a concern. Keeping the immediate audience of this workflow phase, our research team, at the forefront of our mind can help us take steps to structure both mechanistic and analysis code in a useful way.

Mechanistic code, which is designed for repeated use, often employs abstractions by wrapping code into functions that apply the same action repeatedly or stringing together multiple scripts into a computational pipeline. **Unit tests** and so-called **accessor functions** or **getter and setter functions** that extract parameter values from **data structures** or set new values are examples of mechanistic code that might be included in a data-intensive research analysis. Meanwhile, code that is designed to gain statistical insight into distributions or model scientific dynamics using mathematical equations are 2 examples of analysis code. Sometimes, the line between mechanistic code and analysis code can be a blurry one. For example, we might write a looping function to sample our data set repeatedly, and that would classify as mechanistic code. But that sampling may be designed to occur according to an algorithm such as Markov Chain Monte Carlo that is directly tied to our desire to sample from a specific probability distribution; therefore, this could be labeled analysis and mechanistic code. Keep your audience in mind and the reproducibility of your experiment when considering how to present your code.

It is common practice to wrap code that we use repeatedly into functions to increase readability and **modularity** while reducing the propensity for user-induced error. However, the scripts and programming notebooks so useful to establishing a narrative and documenting work in the Refine Phase are set up to be read in a linear fashion. Embedding mechanistic functions in the midst of the research narrative obscures the utility of the notebooks in telling the research story and generally clutters up the analysis with a lot of extra code. For example, if we develop a function to eliminate the redundancy of repeatedly restructuring our data to produce a particular type of plot, we do not need to showcase that function in the middle of a computational notebook analyzing the implications of the plot that is created—the point is the research implications of the image, not the code that made the plot. Then where do we keep the data-reshaping, plot-generating code?

Strategies to structure the more mechanistic aspects of our analysis can be drawn from common software development practices. As our team grows or changes, we may require the same mechanistic code. For example, the same data-reshaping, plot-generating function described earlier might be pulled into multiple computational experiments that are set up in different locations, computational notebooks, scripts, or Git branches. Therefore, a useful approach would be to start collecting those mechanistic functions into their own script or file, sometimes called “helpers” or “utils,” that acts as a supplement to the various ongoing experiments, wherever they may be conducted. This separate script or file can be referenced or “called” at the beginning of the individual data analyses. Doing so allows team members to benefit from collaborative improvements to the mechanistic code without having to reinvent the wheel themselves. It also preserves the narrative properties of team members’ analysis-centric computational notebooks or scripts while maintaining transparency in basic methodologies that ensure project-wide reproducibility. The need to begin collecting mechanistic functions into files separate from analysis code is a good indicator that it may be time for the research team to supplement computational notebooks by using a code editor or IDE for further code development.

Testing scientific software is not always perfectly analogous to testing typical software development projects, where automated **continuous integration** is often employed [17]. However, as we start to modularize our code, breaking it into functions and from there into separate scripts or files that serve specific purposes, principles from software engineering become more readily applicable to our data-intensive analysis. Unit tests can now help us ensure that our mechanistic functions are working as expected, formalizing the “gut checks” that we performed in the Explore Phase. Among other applications, these tests should verify that our functions return the appropriate value, object type, or error message as needed [33]. Formal tests can also provide a more extensive investigation of how “trustworthy” the performance of a particular analysis method might be, affording us an opportunity to check the correctness of our scientific inferences. For example, we could use **control data sets** where we know the result of a particular analysis to make sure our analysis code is functioning as we expect. Alternatively, we could also use a **regression test** to compare computational outputs before and after changes in the code to make sure we haven’t introduced any unanticipated behavior.

### When to transition from the Refine Phase: Going backwards and forwards

Workflows in data science are rarely linear; it is often necessary for researchers to iterate between the Refine and Explore Phases (Fig 1B). For example, while our research team may decide on a computational experimental design to pursue in the Refine Phase, the scope of that design may require us to revisit decisions made during the data processing that was conducted in the Explore Phase. This might mean including additional information from supplementary data sets to help refine our hypothesis or research question. In returning to the Explore Phase, we investigate these potential new data sets and decide if it makes sense to merge them with our original data set.

Iteration between the Refine and Explore Phases is a careful balance. On the one hand, we should be careful not to allow “scope creep” to expand our problem space beyond an area where we are able to develop constructive research contributions. On the other hand, if we are too rigid about decisions made over the course of our workflow and refuse to look backwards as well as forwards, we may risk cutting ourselves off from an important part of the potential solution space.

Data-intensive researchers can once more look to principles within the software development community, such as **Agile** frameworks, to help guide the careful balancing act required to conduct research that is both comprehensive and able to be completed [34,35]. How a team organizes and further documents their organization process can serve as research products themselves, which we describe further in the next phase of the workflow: the Produce Phase.

## Phase 3: Produce

In the previous sections of this paper, we discussed how to progress from the exploration of raw data through the refinement of a research question and selection of an analytical methodology. We also described how the details of that workflow are guided by the breadth of the immediately relevant audience: ourselves in the Explore Phase and our research team in the Refine Phase. In the Produce Phase, it becomes time to make our data analysis camera ready for a much broader group, bringing our research results into a state that can be understood and built upon by others. This may translate to developing a variety of research products in addition to—or instead of—traditional academic outputs like peer-reviewed publications and typical software development products such as computational tools.

### Beyond data analysis: Goals and standards of the Produce Phase

The main goal of the Produce Phase is to prepare our analysis to enter the public realm as a set of products ready for external use, reflection, and improvement. The Produce Phase encompasses the cleanup that happens prior to initially sharing our results to a broader community beyond our team, for example, ahead of submitting our work to peer review. It also includes the process of incorporating suggestions for improvement prior to finalization, for example, adjustments to address reviewer comments ahead of publication. The research products that emerge from a given workflow may vary in both their form and their formality—indeed, some research products, like a code base, might continually evolve without ever assuming “final” status—but each product constitutes valuable contributions that push our field’s scientific boundaries in their own way.

**Fig 2:**
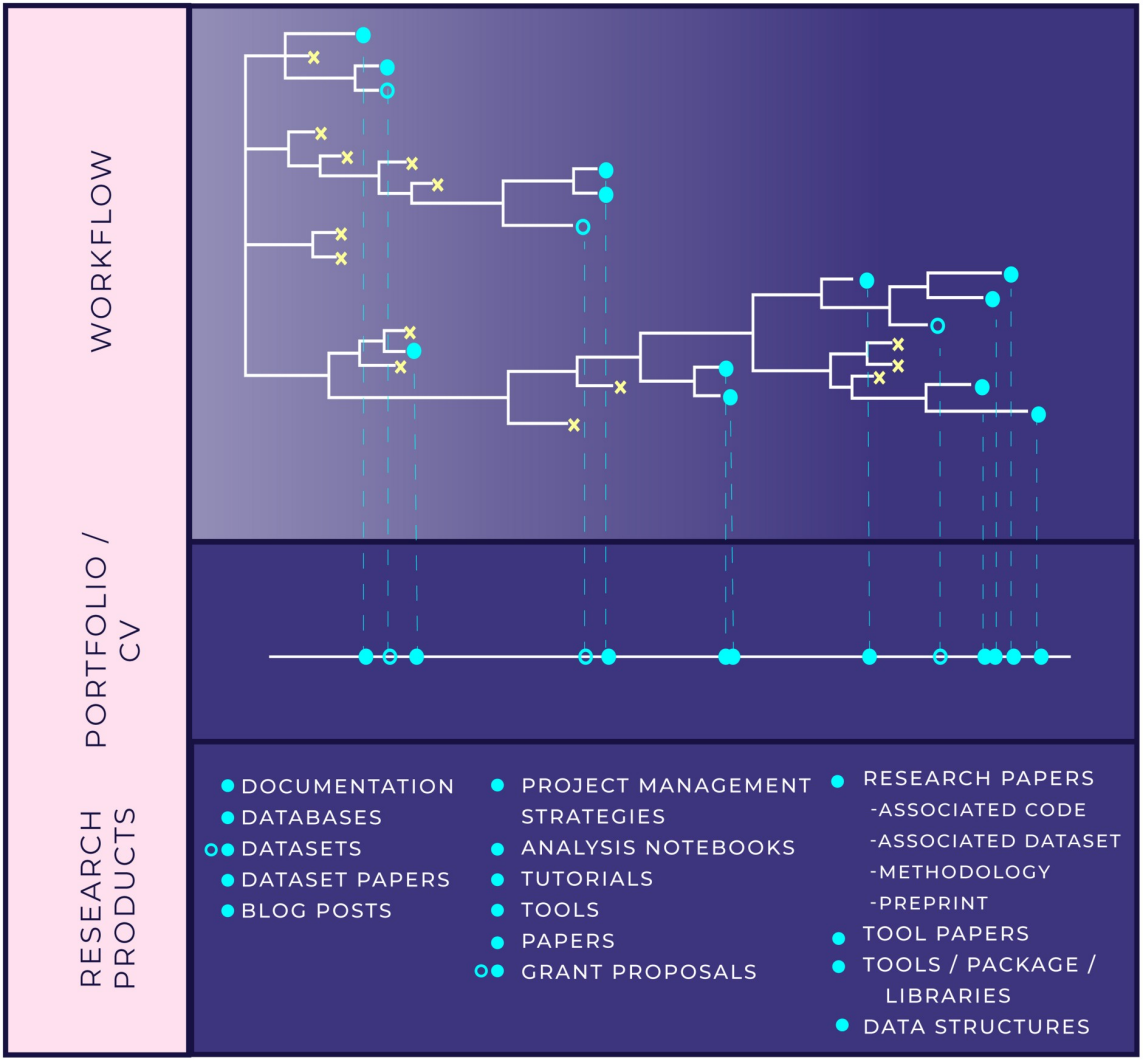
Building our portfolio and CV with ERP. Research products can build off of content generated in either the Explore or the Refine Phase. As they did in Fig 1A, turquoise circles represent potential research products generated as the project develops Closed circles represents research project within scope of project, while open circles represent beyond scope of current project. This figure emphasizes how those research products project onto a timeline and represent elements in our portfolio of work or lines on a CV. The ERP workflow emphasizes and encourages production, beyond traditional, academic research products, throughout the lifecycle of a data-intensive project rather than just at the very end.

Importantly, producing public-facing products over the course of an entire workflow (Fig 2) rather than just at the end of a project can help researchers progressively build their data science research portfolios and fulfill a second goal of the Produce Phase: gaining credit, and credibility, in our domain area. This is especially relevant for junior scientists who are just starting research careers or who wish to become industry data scientists [3]. Developing polished products at several intervals along a single workflow is also instructional for the researcher themselves. Researchers who prepare their work for public assessment from the earliest phases of an analysis become acquainted with the pertinent problem and solution spaces from multiple perspectives. This additional understanding, together with the feedback that polished products generate from people outside ourselves and our immediate team, may furnish insights that improve our approach in other phases of the research workflow.

Building our data science research portfolio requires a method for tracking and attributing the many products that we might develop. One important method for tracking and attribution is the digital object identifier or DOI. It is a unique handle, standardized by the International Organization for Standardization (ISO), that can be assigned to different types of information objects. DOIs are usually connected to metadata, for example, they might include a URL pointing to where the object they are associated with can be found online. Academic researchers are used to thinking of DOIs as persistent identifiers for peer-reviewed publications. However, DOIs can also be generated for data sets, GitHub repositories, computational notebooks, teaching materials, management plans, reports, **white papers**, and preprints. Researchers would also be well advised to register for a unique and persistent digital identifier to be associated with their name, called an ORCID iD (https://orcid.org), as an additional method of tracking and attributing their personal outputs over the course of their career.

A third, longer-term goal of the Produce Phase involves establishing a researcher’s professional trajectory. Every individual needs to gauge how their compendium of research products contribute to their career and how intentional portfolio building might, in turn, drive the research that they ultimately conduct. For example, researchers who wish to work in academia might feel obliged to obtain “academic value” from less traditional research products by essentially reprising them as peer-reviewed papers. But judging a researcher’s productivity by the metric of paper authorship can alter how and even whether research is performed [36]. Increasingly, academic journals are revisiting their publishing requirements [37] and raising their standards of reproducibility. This shift is bringing the data and programming methodologies that underpin our written analyses closer to center stage. Data-intensive research, and the people who produce it, stand to benefit. Scientists—now encouraged, and even required by some academic journals to share both data and code—can publish and receive credit as well as feedback for the multiple research products that support their publications. Questions to ask ourselves as we consider possible research products can be found in Box 2.

### Produce: Products of the Explore Phase

The old adage that one person’s trash is another’s treasure is relevant to the Explore Phase of a data science analysis: Of the many potential applications for a particular data set, there is often only time to explore a small subset. Those applications which fall outside the scope of the current analysis can nonetheless be valuable to our future selves or to others seeking to conduct their own analyses. To that end, the documentation that accompanies data exploration can furnish valuable guidance for later projects. Further, the cleaned and processed data set that emerges from the Explore Phase is itself a valuable outcome that can be assigned a DOI and rendered a formal product of this portion of the data analysis workflow, using outlets like Dryad (http://www.datadryad.org) and Figshare (https://figshare.com/) among others.

Publicly sharing the data set, along with its metadata, is an essential component of scientific transparency and reproducibility, and it is of fundamental importance to the scientific community. Data associated with a research outcome should follow “FAIR” principles of findability, accessibility, interoperability, and reusability. Importantly, discipline-specific data standards should be followed when preparing data, whether the data are being refined for public-facing or personal use. Data-intensive researchers should familiarize themselves with the standards relevant to their field of study and recognize that meeting these standards increases the likelihood of their work being both reusable and reproducible. In addition to enabling future scientists to use the data set as it was developed, adhering to a standard also facilitates the creation of synthetic data sets for later research projects. Examples of discipline-specific data standards in the natural sciences are Darwin Core (https://dwc.tdwg.org) for biodiversity data and EML (https://eml.ecoinformatics.org) for ecological data. To maximize the utility of a publically accessible data set, during the Produce Phase, researchers should confirm that it includes descriptive README files and field descriptions and also ensure that all abbreviations and coded entries are defined. In addition, an appropriate license should be assigned to the data set prior to publication: The license indicates whether, or under what circumstances, the data require attribution.

The Git repositories or computational notebooks that archive a data scientist’s approach, record the process of uncovering coding bugs, redundancies, or inconsistencies and note the rationale for focusing on specific aspects of the data are also useful research products in their own right. These items, which emerge from software development practices, can provide a touchstone for alternative explorations of the same data set at a later time. In addition to documenting valuable lessons learned, contributions of this kind can formally augment a data-intensive researcher’s registered body of work: Code used to actively clean data or record an Explore Phase process can be made citable by employing services like Zenodo to add a DOI to the applicable Git commit. Smaller code snippets or data excerpts can be shared—publicly or privately—using the more lightweight GitHub Gists (https://gist.github.com/). Tools such as Dr.Watson (https://github.com/JuliaDynamics/DrWatson.jl) and Snakemake [23] are designed to assist researchers with organization and reproducibility and can inform the polishing process for products emerging from any phase of the analysis (see [22] for more discussion of reproducible workflow design and tools). As with data products, in the Produce Phase, researchers should license their code repositories such that other scientists know how they can use, augment, or redistribute the contents. The Produce Phase is also the time for researchers to include descriptive README files and clear guidelines for future code contributors in their repository.

Alternative mechanisms for crediting the time and talent that researchers invest in the Explore Phase include relatively informal products. For example, blog posts can detail problem space exploration for a specific research question or lessons learned about data analysis training and techniques. White papers that describe the raw data set and the steps taken to clean it, together with an explanation of why and how these decisions were taken, might constitute another such informal product. Versions of these blog posts or white papers can be uploaded to open-access websites such as arXiv.org as preprints and receive a DOI.

The familiar academic route of a peer-reviewed publication is also available for products emerging from the Explore Phase. For example, depending on the domain area of interest, journals such as *Nature Scientific Data* and *IEEE Transactions* are especially suited to papers that document the methods of data set development or simply reproduce the data set itself. Pedagogical contributions that were learned or applied over the course of a research workflow can be written up for submission to training-focused journals such as the *Journal of Statistics Education*. For a list of potential research product examples for the Explore Phase, see Box 3.

**Box 3. Products**

Research products can be developed throughout the ERP workflow. This box helps identify some options for each phase, including products less traditional to academia. Those that can be labeled with a digital object identifier (DOI) are marked as such.

Potential Products in the Explore Phase

1. Publication of cleaned and processed data set (DOI)
2. Citable GitHub repository and/or computational notebook that shows data cleaning/processing, exploratory data analysis. (e.g., Jupyter Notebook, Knitr, Literate, Pluto, etc.) (DOI)
3. GitHub Gists (e.g., particular piece of processing code)
4. White paper (e.g., explaining a data set)
5. Blog post (e.g., detailing exploratory process)
6. Teaching/training materials (e.g., data wrangling)
7. Preprint (e.g., about a data set or its creation) (DOI)
8. Peer-reviewed publication (e.g., about a curated data set) (DOI)

Potential Products in the Refine Phase

1. White paper (e.g., explaining preliminary findings)
2. Citable GitHub repository and/or computational showing methodology and results (DOI)
3. Blog post (e.g., explaining findings informally)
4. Teaching/training materials (e.g., using your work as an example to teach a computational method)
5. Preprint (e.g., preliminary paper before being submitted to a journal) (DOI)
6. Peer-reviewed publication (e.g., formal description of your findings) (DOI)
7. Grant application incorporating the data management procedure
8. Methodology (e.g., writing a methods paper) (DOI)
9. A software tool
  - This might include a package, a library, or an interactive web application.
  - See Box 4 for further discussion of this potential research product.

### Produce: Products of the Refine Phase

In the Refine Phase, documentation and the ability to communicate both methods and results become essential to daily management of the project. Happily, the implementation of these basic practices can also provide benefits beyond the immediate team of research collaborators: They can be standardized as a Data Management Plan or Protocol (DMP). DMPs are a valuable product that can emerge from the Refine Phase as a formal version of lessons learned concerning both research and team management. This product records the strategies and approaches used to, for example, describe, share, store, analyze, and preserve data.

While DMPs are often living documents over the course of a research project, evolving dynamically with the needs or restrictions that are encountered along the way, there is great utility to codifying them either for our team’s later use or for others conducting similar projects. DMPs can also potentially be leveraged into new research grants for our team, as these protocols are now a common mandate by many funders [38]. The group discussions that contribute to developing a DMP can be difficult and encompass considerations relevant to everything from team building to research design. The outcome of these discussions is often directly tied to the constructiveness of a research team and its robustness to potential turnover [38]. Sharing these standards and lessons learned in the form of polished research products can propel a proactive discussion of data management and sharing practices within our research domain. This, in turn, bolsters the creation or enhancement of community standards beyond our team and provides training materials for those new to the field.

As with the research products that are generated by the Explore Phase, DMPs can lead to polished blog posts, training materials, white papers, and preprints that enable researchers to both spread the word about their valuable findings and be credited for their work. In addition, peer-reviewed journals are beginning to allow the publication of DMPs as a formal outcome of the data analysis workflow (e.g., *Rio Journal*). Importantly, when new members join a research team, they should receive a copy of the group’s DMP. If any additional training pertinent to plans or protocols is furnished to help get new members up to speed, these materials too can be polished into research products that contribute to scientific advancement. For a list of potential research product examples for the Refine Phase, see Box 3.

### Produce: Traditional research products and scientific software

By polishing our work, we finalize and format it to receive critiques beyond ourselves and our immediate team. The scientific analysis and results that are born of the full research workflow—once documented and linked appropriately to the code and data used to conduct it—are most frequently packaged into the traditional academic research product: a peer-reviewed publication. Even this product, however, can be improved upon in terms of its reproducibility and transparency thanks to software development tools and practices. For example, papers that employ literate programming notebooks enable researchers to augment the real-time evolution of a written draft with the code that informs it. A well-kept notebook can be used to outline the motivations for a manuscript and select the figures best suited to conveying the intended narrative, because it shows the evolution of ideas and the mathematics behind each analysis along with—ideally—brief textual explanations.

Peer-reviewed papers are of primary importance to the career and reputation of academic researchers [39], but the traditional format for such publications often does not take into account essential aspects of data-intensive analysis such as computational reproducibility [40]. Where strict requirements for reproducibility are not enforced by a given journal, researchers should nonetheless compile the supporting products that made our submitted manuscript possible—including relevant code and data, as well as the documentation of our computational tools and methodologies as described in the earlier sections of this paper—into a research compendium [37,41–43]. The objective is to provide transparency to those who read or wish to replicate our academic publication and reproduce the workflow that led to our results.

In addition to peer-reviewed publications and the various alternative research products described above, some scientists may choose to revisit the scripts developed during the Explore or RefinePhases and polish that code into a traditional software development product: a computational tool, also called a **software tool**. A computational tool can include libraries, packages, collections of functions, or data structures designed to help with a specific class of problem. Such products might be accompanied by repository documentation or a full-fledged methodological paper that can be categorized as additional research products beyond the tool itself. Each of these items can augment a researcher’s body of citable work and contribute to advances in our domain science.

One very simple example of a tool might be an interactive web application built in RShiny (https://shiny.rstudio.com/) that allows the easy exploration of cleaned data sets or demonstrates the outcomes of alternative research questions. More complex examples include a software package that builds an open-source analysis pipeline or a data structure that formally standardizes the problem space of a domain-specific research area. In all cases, the README files, docstrings, example vignettes, and appropriate licensing relevant to the Explore phase are also a necessity for open-source software. Developers should also specify contributing guidelines for future researchers who might seek to improve or extend the capabilities of the original tool. Where applicable, the dynamic equations that inform simulations should be cited with the original scientific literature where they were derived.

The effort to translate reproducible scripts into reusable software and then to maintain the software and support users is often a massive undertaking. While the software engineering literature furnishes a rich suite of resources for researchers seeking to develop their own computational tools, this existing body of work is generally directed toward trained programmers and software engineers. The design decisions that are crucial to scientists—who are primarily interested in data analysis, experiment **extensibility**, and result reporting and inference—can be obscured by concepts that are either out of scope or described in overtly technical jargon. Box 4 furnishes a basic guide to highlight the decision points and architectural choices relevant to creating a tool for data-intensive research. Domain scientists seeking to wade into computational tool development are well advised to review the guidelines described in Gruning and colleagues [2] in addition to more traditional software development resources and texts such as Clean Code [44], Refactoring [45], and Best Practices in Scientific Computing [24].

**Box 4. Tool development guide**

Creating a new software tool as the polished product of a research workflow is nontrivial. This box furnishes a series of guiding questions to help researchers think through whether tool creation is appropriate to project goals, domain science needs, and team member skill sets.

1. Should I make a tool?
  - Does a tool in this space already exist that can be used to provide the functionality/answer the research question of interest?
  - Does a new tool add to the body of scientific knowledge?
    - Does it formalize our research question?
    - Does it extend/allow extension of investigative capabilities beyond the research question that our existing script was developed to ask?
  - Does creating a tool advance our personal career goals or augment a desired/necessary skill set?
  - Do we have the resources required to develop a new tool?
    - Time?
    - Funding (if applicable)?
    - Domain expertise?
    - Programming expertise?
    - Collaborative research partners with either time, funding, or relevant expertise?
    - Will the process of creating the new tool be valued/helpful for your career goals?
2. What tool should we create?
  - Should we build on an existing tool or make a new one?
  - What is the scope of a new tool?
    - What research area is it designed for?
    - Who is the envisioned end user? (e.g., scientist inside our domain, scientist outside our domain, policy maker, member of the public)
    - What is the goal of the end user? (e.g., analysis of raw inputs, explanation of results, creation of inputs for the next step of a larger analysis)
3. How should we structure the tool?
  - Language choice
    - What are field norms?
    - Is it accessible (free, open source)?
  - Data structures and types
    - What is the likely form and type of data input to our tool?
    - What is the desired form and type of data output from our tool?
    - Are there preexisting structures that are useful to emulate, or should we develop our own?
  - Platform or framework
    - Is there an existing package that provides basic structure or building block functionalities necessary or useful for our tool, such that we do not need to reinvent the wheel?

## Conclusions

Defining principles for data analysis workflows is important for scientific accuracy, efficiency, and the effective communication of results, regardless of whether researchers are working alone or in a team. Establishing standards, such as for documentation and unit testing, both improves the quality of work produced by practicing data scientists and sets a proactive example for fledgling researchers to do the same. There is no single set of principles for performing data-intensive research. Each computational project carries its own context—from the scientific domain in which it is conducted, to the software and methodological analysis tools we use to pursue our research questions, to the dynamics of our particular research team. Therefore, this paper has outlined general concepts for designing a data analysis such that researchers may incorporate the aspects of the ERP workflow that work best for them. It has also put forward suggestions for specific tools to facilitate that workflow and for a selection of nontraditional research products that could emerge throughout a given data analysis project.

Aiming for full reproducibility when communicating research results is a noble pursuit, but it is imperative to understand that there is a balance between generating a complete analysis and furnishing a 100% reproducible product. Researchers have competing motivations: finishing their work in a timely fashion versus having a perfectly documented final product, while balancing how these trade-offs might strengthen their career. Despite various calls for the creation of a standard framework [7,46], achieving complete reproducibility may go far beyond the individual researcher to encompass a culture-wide shift in expectations by consumers of scientific research products, to realistic capacities of version control software. The first of these advancements is particularly challenging and unlikely to manifest quickly across data-intensive research areas, although it is underway in a number of scientific domains [26]. By reframing what a formal research product can be—and noting that polished contributions can constitute much more than the academic publications previously held forth as the benchmark for career advancement—we motivate structural change to data analysis workflows.

In addition to amassing outputs beyond the peer-reviewed academic publication, there are increasingly venues for writing less traditional papers that describe or consist solely of a novel data set, a software tool, a particular methodology, or training materials. As the professional landscape for data-intensive research evolves, these novel publications and research products are extremely valuable for distinguishing applicants to academic and nonacademic jobs, grants, and teaching positions. Data scientists and researchers should possess numerous and multifaceted skills to perform scientifically robust and computationally effective data analysis. Therefore, potential research collaborators or hiring entities both inside and outside the academy should take into account a variety of research products, from every phase of the data analysis workflow, when evaluating the career performance of data-intensive researchers [47].
